# A new community for those involved and interested in diagnosis and prognosis

**DOI:** 10.1186/s41512-016-0007-5

**Published:** 2017-02-08

**Authors:** Karel G. M. Moons, Nancy Cook, Gary Collins

**Affiliations:** 1 Julius Center for Health Sciences and Primary Care, Julius Center, University Medical Center Utrecht, Utrecht, Netherlands; 2 Brigham and Women’s Hospital, Harvard Medical School, Boston, MA USA; 3 Harvard T.H. Chan School of Public Health, Boston, MA USA; 4 Centre for Statistics in Medicine, NDORMS, University of Oxford, Oxford, UK

## Abstract

Why do we need a new journal titled *Diagnostic and Prognostic Research* in the year 2017, in an era where communication has shifted towards instant messaging via avenues such as Twitter, blogs and Facebook and where we already have tens of thousands of biomedical journals, producing millions of papers, editorials and letters each year?

## Editorial

A journal entitled *Diagnostic and Prognostic Research*—why do we actually need this in the year 2017, in an era where communication has shifted towards short messages via Twitter, blogs and Facebook and where we already have 10,000s of biomedical journals, producing millions of papers, editorials and letters each year?

The answers are easy. Even though making a diagnosis in an individual and estimating someone’s prognosis form the essentials of medical care, a journal with this specific focus is interestingly enough still lacking. The domain of applied medical research, as opposed to the more fundamental biomedical research, has predominantly focused on evaluation of therapeutic or preventive interventions (intervention research) and of studies aimed at unravelling the causes of diseases (aetiology research). Numerous papers and books have been written on how to design, write protocols, conduct, analyse and report therapeutic intervention studies, with a focus on randomised trials. Similarly, many publications have addressed how to study causality or causal associations between risk factors and diseases. This applies not only to primary (intervention or aetiological) studies but also to meta-research, such as systematic reviews and meta-analyses, of such studies.

In contrast, although interest in evaluation of diagnostics and prognostics has clearly increased in the past two decades across many clinical areas, there is relatively still little guidance on how to design, develop protocols, and conduct, analyse and report research aimed at quantifying the safety, accuracy, effectiveness and cost-effectiveness of diagnostic, prognostic, monitoring or screening tools. These tools include not only tests or (bio)markers but also so-called prediction models, decision aids, apps or any other method used to improve diagnosis, including screening and early diagnosis, and to improve prognosis, including monitoring of diseases and therapeutic effectiveness. As a consequence, numerous reviews have shown that the literature on diagnostic and prognostic evaluations suffers from poor design, conduct, statistical analysis and reporting.

Published research from (observational) diagnostic and prognostic studies outnumbers that from randomized intervention trials [1]. Yet due to the lack of mandatory requirement for registering observational research, compared to therapeutic intervention trials, their findings are likely more subject to data dredging, false-positive and spurious findings, and to selective reporting including non-publication. Calls have been made in recent years for (observational) diagnostic and prognostic research to be protocol-driven and registered [2–4]. Although pre-registration of specific hypothesis-based research is no guarantee of removing all the various biases in diagnostic or prognostic research, it will help identify discrepancies or unplanned analyses whilst improving transparency, including the publication of results irrespective of whether they met some nominal level of statistical significance.

Also, compared to drug innovations, innovations (e.g. tests, markers, models or apps) aimed at improving diagnosis, prognosis, screening or monitoring are faced with a very different legislation, regulatory process and market access. Drug research is dominated and controlled by strict regulatory processes, even prescribing which types of studies need to be done in which order, what needs to be pre-registered and what needs to be reported in protocols and reports, before a drug is allowed to the market. This is much less—or not at all—the case for the vast majority of diagnostic and prognostic innovations. Many prognostic and diagnostic innovations (e.g. biomarker assessments, prediction models or decision apps) do not require any formal evaluation before being allowed market access or being implemented in medical guidelines or indeed in daily healthcare. This in an era where the number of newly developed or discovered diagnostic and prognostic tools almost increases per day. This involves tools that are intended not only for end users including healthcare professionals in hospitals, primary care, nursery homes and home care but also for patients or individuals themselves to enhance eHealth or mHealth.

All the above means that the medical community with respect to diagnostics and prognostics is very volatile, less guided and largely a self-regulating and self-controlling community. This puts an even bigger demand on studies and reports of diagnostics and prognostics: only when appropriately designed, protocol-driven, well conducted and analysed, and transparently reported can others reproduce or validate previous findings and use the test, marker, model or app in practice. *Diagnostic and Prognostic Research* aims to contribute to improving on all of these aspects.

### Personalized or precision medicine and big data

Personalized or precision medicine is all over the place, and we are entering the era designated by the buzzword ‘big data’. Personalized, precision or risk-based medicine aims to administer and tailor therapeutic and preventive interventions based on expected risks or benefits of the treated person. The individual’s expected risks and benefits are almost always determined by (early) diagnostic or prognostic and monitoring information. This information ranges from results of ‘omics tests’ and companion diagnostics, to imaging test results and biomarkers measured in blood or stool, to personal characteristics such as age, gender and medical history. Moreover, such diagnostic and prognostic information can be used in isolation or in combination in so-called diagnostic and prognostic prediction models. In other words, personalized, precision or risk-based medicine can only flourish when we design, protocolize, conduct, analyse and report proper diagnostic and prognostic research.

Big data seems a phrase with big meanings that now appears everywhere—so here as well. It seems that ‘everyone’ uses the term, whereas nobody seems to be able to really define it in a single sentence. By no means do we aim to settle this here. But we do believe that a key application of (analyses of) big data is in diagnostic and prognostic research. The combination of multiple (types of) data and data sources of individuals allows one to discover and validate diagnostic and prognostic pieces of information to enhance personalized, precision or risk-based medicine.

It comes to no surprise that we thus believe it is timely for a journal called *Diagnostic and Prognostic Research*.

### Goal and focus

Our goal is to attract researchers, healthcare providers, patients and other stakeholders interested or involved in the evaluation and use of tests, markers, models, apps or any other tool intended for medical diagnosis, prognosis, screening and monitoring (Table 1). Our aim is to enhance the implementation and use of safe and useful tools by the targeted end users, and to discourage the use of redundant and useless tools. We hope to become a resource and virtual meeting place for authors, healthcare providers, patients, regulatory agencies and other stakeholders involved and interested in medical diagnosis and prognosis, providing a single site with comprehensive state-of-the-art information on all issues addressed above. To this aim, we will use several media, e.g. blogs and Twitter, besides just publishing reports.

**Table 1:** Mission statement of *Diagnostic and Prognostic Research*

• *Diagnostic and Prognostic Research* welcomes all diagnostic and prognostic research addressing studies on the evaluation of all types of medical tests, markers, prediction models, decision tools and apps, regardless of the clinical domain. • *Diagnostic and Prognostic Research* also welcomes studies on tests used for screening or early diagnosis and for monitoring disease progress or therapy response. • *Diagnostic and Prognostic Research* welcomes submissions with a focus on disseminating empirical primary studies and systematic reviews (including meta-analyses) as well as articles on methodology, protocols and commentaries addressing diagnostic and prognostic studies. • *Diagnostic and Prognostic Research* believes all well-conducted diagnostic and prognostic research should be published, regardless of their outcome.	

To meet this aim we focus on applied studies on the safety and value of any specific diagnostic, prognostic, monitoring or screening tool in a specific context, regardless of the type of tool, the intended use or target users of the tool, the medical context or domain, the study design (e.g. observational or randomized), the data source (e.g. prospective study, analysis of data of a previous study or of registry data) and the data analytical approach (e.g. regression techniques or machine learning) that is addressed.

We also focus on guidance and theoretical papers or commentaries addressing specific methods, conduct, reporting standards, dissemination and implementation of diagnostic and prognostic research results. We feel it is our obligation to help define and indeed set the standards of valid and useful studies and of complete and transparently reported diagnostic and prognostic study results. Numerous reviews have concluded that standards of scientific conduct and reporting of diagnostic and prognostic studies need to be raised to enhance reproducibility and the applicability of safe and clinically valuable diagnostics, prognostics, monitoring and screening tools into medical practice. Accordingly, we require authors to adhere to various reporting guidelines, including STARD [5], REMARK [6], GRIPS [7] and TRIPOD [8, 9].

In addition to primary studies on specific tests, markers, models or any other tool, and to methods and guidance reports for such studies, we also focus on meta-research of diagnostic and prognostic studies. This may involve meta-studies using aggregate data or results from previous reports, as well as meta-studies using individual participant data or other types of big data sources.

To further improve the visibility and transparency of diagnostic and prognostic research, we also aim to be the first journal that actively encourages investigators and other stakeholders to publish protocols of planned studies—both primary and meta-studies—to enhance the transparency and replication (when needed) of such studies.

### Current issue

The first issue that kick-starts our new journal includes four papers.

Our first paper uses data from two primary care cohorts to illustrate how the magnitude of predictor-outcome associations and prediction model performance can differ depending on the moment at which the prediction is made. With findings replicated in two different datasets, the authors highlight the need for well-conducted external validation of prognostic and diagnostic prediction models and stress the importance to consider differences between model validation and model development studies.

The second paper includes a systematic review to identify all studies that developed or validated models for predicting the risk of developing gestational diabetes in the first trimester of pregnancy. The authors conclude that many of the studies were of moderate to low quality, with a dearth of model validation studies. A lack of external validation, and absence of a head-to-head comparison of these models, leads to an unclear picture as to which, if any, of these models show promise for predicting gestational diabetes.

Staying on the topic of validation, our third paper focuses on methods to validate and update models that predict multicategory outcomes based on multinomial regression. The authors illustrate how to validate and update multinomial models to predict the outcome of pregnancies of unknown location.

Finally, our fourth and more technical paper focuses on the use of so-called benefit-based versus value-based strategies in the evaluation of (diagnostic) tests. The pros and cons of both strategies used for deciding to bring a test into use are illustrated.

### About the journal

*Diagnostic and Prognostic Research* is an international open-access journal that provides broad access to researchers and readers from a variety of backgrounds, fields and countries. We have assembled an outstanding Editorial Board of internationally recognized researchers and healthcare providers who are experts in this type of research and a team of dedicated Associate Editors who are widely acknowledged in the field. We aim to set best-practice standards in evaluations of tests, markers, models or any other tool used for diagnosis, prognosis, screening and monitoring. We hope to move the field forward to attract researchers, healthcare providers, patients and other stakeholders involved in evaluating the safety, accuracy, effectiveness and cost-effectiveness of such tools to enhance their uptake by the intended users and in the intended contexts.

The journal will predominantly be an academic journal publishing papers but with a community focus. We want the journal to be a platform for sharing innovative and recent developments in the evaluation of diagnostic and prognostic tools, and invite stakeholders in the field to submit Commentary articles on debated or topical issues, and use the journal as a platform for advancing the field. As a journal we also support blogs on articles published in the journal and Q&As, and the journal falls within the @MedicalEvidence twitter account, which covers BioMed Central’s applied-methodology journals.

We hope you find the journal to be interesting and useful, and a valuable resource. We encourage you to submit your own work in this area, whether it be applied, methodological, commentary or inquiring. Detailed information about the journal can be found on our website at diagnprognres.biomedcentral.com, and if you have any specific questions, please contact our Editorial Office: diagnprognres@biomedcentral.com.
